# Patent-Based Technological Overview of Propolis–Cyclodextrin Inclusion Complexes with Pharmaceutical Potential

**DOI:** 10.3390/pharmaceutics17070898

**Published:** 2025-07-11

**Authors:** Salvana Costa, Ighor Costa Barreto, Nataly Gama, Kathylen Santos, Cleomárcio Miguel de Oliveira, Isabela Silva Costa, Monique Vila Nova, Ruane Santos, Arthur Borges, José Marcos Teixeira de Alencar Filho, Ticiano Gomes do Nascimento

**Affiliations:** 1 Institute of Pharmaceutical Science, Federal University of Alagoas, Maceio 57072-970, Alagoas, Brazil; nataly.gama@icf.ufal.br (N.G.); kathylen.santos@icf.ufal.br (K.S.); monique.nova@icf.ufal.br (M.V.N.); ruane.santos@icf.ufal.br (R.S.); ticiano@icf.ufal.br (T.G.d.N.); 2 Superintendency of Environment and Infrastructure, Federal University of Bahia, Salvador 40170-110, Bahia, Brazil; ighor.barreto@ufba.br; 3 Pharmacy Department, Irecê College, Irecê 40900-000, Bahia, Brazil; ofarmaceuticomiguel@gmail.com (C.M.d.O.); isabelasilvacosta4@gmail.com (I.S.C.); 4 Technology Center, Federal University of Alagoas, Maceio 57072-970, Alagoas, Brazil; arthurltfb@gmail.com; 5 Pharmacy College, Federal University of Bahia, Salvador 40170-110, Bahia, Brazil; josealencar@ufba.br

**Keywords:** propolis, cyclodextrins, polysaccharides, drug delivery systems

## Abstract

**Background/objectives:** Propolis, known for its medicinal properties, faces challenges in pharmaceutical applications due to its low aqueous solubility, attributed to its resinous and hydrophobic nature. This limits oral administration, reducing its bioavailability and pharmacological activities. To overcome these barriers, cyclodextrins (CDs), cyclic oligosaccharides, are widely studied as carrier systems that enhance the solubility and bioavailability of propolis and other nonpolar compounds. This study aimed to review patents that developed innovative therapeutic approaches to improve the physicochemical and biological properties of propolis through complexation with CDs. **Methods:** Active and application patents registered over the last 17 years were searched across multiple databases, resulting in the selection of eight inventions for detailed analysis. **Results:** These patents highlight therapeutic applications of propolis–CD systems for conditions such as diabetes and skin and gastrointestinal cancers, as well as antimicrobial, immunostimulant, and antioxidant effects. Additionally, novel extraction processes free of organic solvents, including nanometric-scale powder extracts, are described. **Conclusions:** Findings from scientific articles support the patent data, demonstrating that CD complexation significantly enhances the solubility and therapeutic efficacy of propolis. Thus, these patents present an innovative and promising strategy for developing propolis-based pharmaceutical products.

## 1. Introduction

In an increasingly globalized world, innovating in terms of scientific and technological subjects is a key competitive advantage. In this scenario, being capable of anticipating trends and approaches in an innovative way is essential. Mayerhoff [1] highlights that technological prospecting studies rule strategic decisions and reveal promising avenues. A valuable tool for tracking technological trends is the analysis of patent databases, which provide insights into the current state of technology, from patented products to processes. This information is important to keep organizations up-to-date and competitive in a highly dynamic global scenario where innovation is a constant [2].

Propolis, known as “bee glue”, is a resinous mixture mainly obtained from honeybees of the species *Apis mellifera* Linnaeus (Apidae) and is known for its medicinal properties [3,4]. Bees add their own salivary secretions and enzymes to the mixture, resulting in a complex and unique chemical composition that is an essential component for the hive’s survival and health [5].

Its chemical composition can be influenced by local plant sources, seasonality, climate, and bee subspecies [6]. This results in several types of propolis, each with its own chemical fingerprint and characteristics. Therefore, the chemical standardization and identification of botanical sources are crucial for its characterization. In addition, a multiple marker evaluation strategy may be a promising approach to assess the quality of propolis [7].

Although the chemical composition of propolis is very complex and variable due to the factors mentioned above, it generally consists of a plant resin and balm (up to 60 to 70%); pollen; beeswax (<10% to 87%); volatile organic compounds (<1% to 3%); and other classes of compounds such as flavonoids and other plant phenolics, terpenoids, aromatic acids and their esters, aliphatic acids and their esters, amino acids, carbohydrates, vitamins (B1, B2, B6, C and E); and minerals (calcium, aluminum, cesium, iron, antimony, copper, lanthanum, mercury, manganese, nickel, silver, vanadium, and zinc) [7,8].

Flavonoids, terpenoids, phenolic acids, and other polyphenols are involved in its biological properties and therapeutic potential, such as antimicrobial, immunostimulant, antioxidant, anti-inflammatory, cytotoxic, antiviral, anticancer, hepatoprotective, anesthetic, healing, and anticarcinogenic effects [9,10].

Nevertheless, regarding its application in pharmaceutical industries, an obstacle faced is its low aqueous solubility, because propolis is resinous and rich in hydrophobic substances, which limits its oral administration and reduces its bioavailability and pharmacological activities [10,11,12,13]. Therefore, a family of cyclic oligosaccharides composed of six or more glucose units linked by α-1,4 bonds, such as cyclodextrins (CDs), can be used to overcome this low solubility and improve the bioavailability of flavonoids and many other nonpolar compounds [14,15,16,17].

CDs have hydrophobic cavities with a hydrophilic exterior and are versatile carriers for many active hydrophobic compounds in water through some non-covalent interactions, including hydrophobic interactions, electronic effects, van der Waals forces, hydrogen bonding, and steric factors [18,19]. Adding complexes of CDs with propolis improves its solubility, stability, and biological activity, making it possible to include these complexes in different types of preparations [20,21,22].

In view of the scientific progress observed in the literature on the possibility of encapsulating propolis in cyclodextrin, overcoming the limitations of the direct use of this versatile product of natural origin, this work aimed to search granted and active patents of inclusion complexes developed for the transport of propolis, understanding the current state of use of this technology for pharmaceutical purposes and to project the perspectives of scientific investment in the improvement of this transport system for propolis.

## 2. Method

This study was a technological prospection, with an exploratory and descriptive nature, of recent patent applications, covering from 2007 to 2024. The work sought to relate propolis and cyclodextrins, and their pharmaceutical applications, performing a detailed qualitative and quantitative analysis of the evolution of patent applications. The following patent offices were considered as search sources: World Intellectual Property Organization (WIPO-PATENTSCOPE), The Lens, LATIPAT, and INPI. The descriptors were “propolis”, “cyclodextrins”, and “complex inclusion”. Depending on the database, they were searched in English, Spanish, and Portuguese, and their appearance in the abstract, title, or full description was considered using Boolean connectors “AND” and “*”, among other specific search system drivers.

To include patents, some unique criteria were set, like the inclusion of pharmaceutical patents considering the International Patent Classification (IPC) A61K codes (medical, dental, or toilet preparations), the legal status of the patent (active only), and the type of documents (granted patents and patent applications). For further review, documents were excluded if they were duplicates, if they were outside the focus, or if they were not available in full.

## 3. Results

*Propolis and Cyclodextrin Patents*

**Figure 1:**
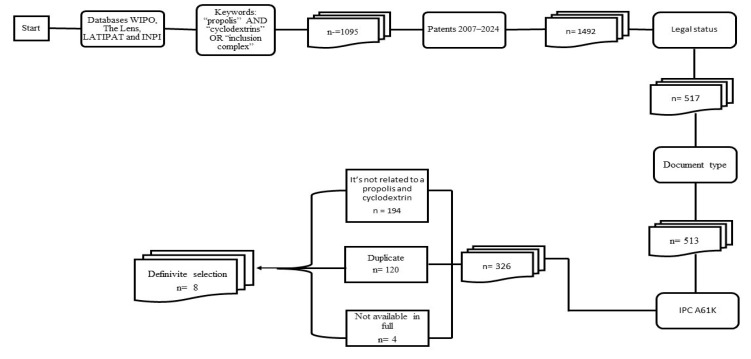
Flowchart of patent searching, filtering, and selection.

The primary database search resulted in the initial identification of 1095 patents, selecting just those published between 2007 and 2024 (n = 1492). Patents published in earlier years were not included because they were considered too old. The following filtering criteria were applied: active legal status (n = 517), document type (n = 513), and IPC classification A61K (n = 326). At this point, 120 were excluded due to duplication, 194 for being irrelevant, and 4 because they were not available in full, leaving 8 patents for full reading. Thus, eight patents were selected for analysis (Figure 1).

**Figure 2:**
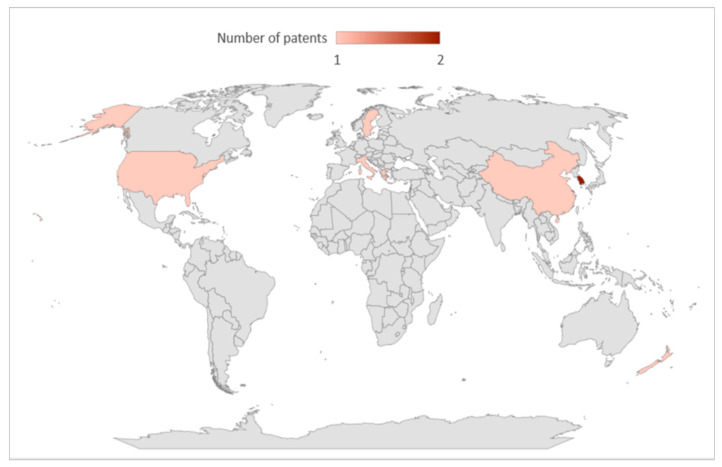
Global distribution of filed patents.

Table 1 summarizes the most important information about patents presented in this study. They were patented by private companies and independent inventors. It is interesting to note that three of the patents filed discuss the therapeutic use of propolis carrier systems with cyclodextrin in their text. The majority of these products are indicated mainly for cancer treatment (2); antimicrobial and immunostimulant activities (1); and improving the stability, solubility, and properties of propolis bioactive compounds (5). Regarding patent filing countries, the United States of America, New Zealand, Italy, Sweden, Greece, and China were identified with one patent filed in each; South Korea appeared with two patents filed (Figure 2).

## 4. Discussion

### 4.1. Binary and Ternary Inclusion Complexes

In general, cyclodextrins form binary complexes that serve as efficient carriers for drugs and natural compounds with low aqueous solubility, improving their dissolution rate and, consequently, their bioavailability [16,31]. Szente et al. [32] reported the successful development of the first binary complex of β-CD with propolis, which exhibited significantly enhanced stability under thermal and alkaline conditions, representing a promising strategy for delivering unstable, poorly soluble compounds found in this natural extract. Junaković et al. [33] further demonstrated that the HP-β-CD complex containing propolis maintained high polyphenolic bioavailability throughout all three simulated digestion phases. Notably, after the intestinal phase with centrifugation, the bioavailability of total polyphenols, flavonols/flavones, and flavanones/dihydroflavonols was 0.02 to 8.86 times greater than that observed in the dialysis phase, reinforcing the relevance of this model as a closer approximation to physiological conditions. These findings support the well-established role of inclusion complexes in enhancing the solubility and bioavailability of polyphenol groups [17]. Subsequently, several studies explored the biological activities of inclusion complexes of propolis with β-CD [34,35,36], as well as of individual polyphenolic compounds complexed with various types of cyclodextrins, including α-, β-, and γ-cyclodextrin and their derivatives [37,38,39].

Patent number 1 [23] (Table 1) registered a product consisting of New Zealand propolis encapsulated with γ-CD effective against the colon cancer cell lines HCT116/HCT116-R (butyrate-resistant). The in vitro antiproliferative activity of the propolis–γ-CD complex alone and with several other propolis fractions was determined against these cell lines. However, only the propolis–γ-CD complex was considered effective in inducing apoptosis. This same work was further developed and published by Catchpole et al. [40], who investigated the anti-gastrointestinal cancer activity of New Zealand propolis encapsulated with α-CD, β-CD, and γ-CD. The New Zealand propolis complexes inhibited the proliferation of four human gastrointestinal cancer cell lines, with the degree of inhibition increasing with an increase in exposure time.

New Zealand propolis has a huge caffeic acid phenethyl ester (CAPE) content, which has been reported in the literature to have activity against several cancer cell lines, mainly when complexed with γ-CD [41] and even more effective and better encapsulated compared to α- and β-CD [37]. Wadhwa et al. [42] demonstrated that CAPE mediated mortalin downregulation and tumor suppressor protein p53 activation, which are specific to cancer cells. In addition, it demonstrated activity against cancer cell lines, like breast carcinoma (MCF-7 and MDA-MB-231), fibrosarcoma (HT1080), melanoma (G361), osteosarcoma (U2OS), and human lung carcinoma (A549). It was noted that caffeic acid degradation by secreted esterases turned CAPE unstable in the culture medium. Nevertheless, the complex with γ-CD displayed potent efficacy in antitumor and antimetastasis assays, proving successful both in vitro and in vivo, whether administered intraperitoneally or orally.

In addition, the Japanese company CycloChem Bio showed that the propolis inclusion complex with γ-CD was superior to α- and β-CD in terms of complex dispersibility in water, reduction in pungency, and retention of the main bioactive compounds of propolis [37]. It was also shown that orally administered CAPE encapsulated in γ-CD was more effective in slowing the growth of fibrosarcoma cancers induced in rats than CAPE alone [43].

The better activity of NZ/γ-CD complexes compared to α- and β-CD against these cancer cell lines likely refers to the larger hydrophobic internal cavity of γ-CD, which enables the retention of a greater amount and larger active molecules [11], having a non-coplanar and more flexible structure that gives it a much higher solubility (232 g/L, 25 °C) than α-CD (145 g/L, 25 °C) and β-CD (18.5 g/L, 25 °C) [32]. In addition, it has the ability to increase the aqueous solubility of poorly soluble drugs (i.e., BCS class II and IV drugs), resulting in improved drug dissolution, permeation, and bioavailability [15]. As a result, it has a more favorable pharmacokinetic and toxicological profile [44]. In this way, γ-CD forms more efficient complexes and results, with a positive impact on the release and bioavailability of active compounds.

Patent No. 2 [24] registered the development of a product for the treatment and prevention of skin cancer (basal cell carcinoma, squamous cell carcinoma, and melanoma) and the improvement of skin health. The inventors developed nine formulations (CD1 to CD9), varying mainly in concentration, the type of propolis, and the type of CD (α, β, and γ). For anticancer evaluation in human melanoma, the results of this test showed that the γ-CD complex of New Zealand (NZ) propolis (CD1) presented similar results to the dry extract of NZ propolis, both at the same concentration of 50 µg/mL, exhibiting 21% and 26% antiproliferative activity, respectively. In contrast, the Brazilian green propolis tested at 50 µg/mL demonstrated a low antiproliferative activity (12%); consequently, its γ-CD complex (CD4) did not exhibit a significant antiproliferative activity. However, the inclusion of complexes α-CD (CD2) and β-CD (CD3) with NZ propolis, in comparison with the positive control 5-fluorouracil, a common treatment of multiple neoplasms, presented higher antiproliferative activity compared to the same amount of propolis extract, 35% and 42% antiproliferative activity, respectively.

The superior antiproliferative activity of α- and β-CD over γ-CD could be caused by the higher concentration of propolis solids, which consequently resulted in a greater presence of flavonoids and other compounds with enhanced anticancer effects. For example, pinocembrin and benzyl ferulate are the strongest of these compounds, followed by tectocrisin, l,l-dimethylallyl caffeate, pinobanksin-3-O-acetate, and 3-methyl-3-butenyl caffeate. Pinocembrin and benzylferulate completely killed all melanoma cells at a concentration of 200 μg/mL. Tectocrisin was also a very potent inhibitor of proliferation at 86.5%, as were 1,1-dimethylallylcaffeate at 73.5% and pinobanksin-3-o-actetate at 61.4%. 3-Methyl-3-butenylcaffeate was also a potent inhibitor of proliferation at 46.8%, although its concentration was only 20 μg/mL. CAPE at 200 μg/mL was a moderate-to-strong inhibitor of proliferation at 49.4%. The α- and β-CD complex contained better encapsulation rates than pinocembrin and pinobanksin-3-O-acetate. Thus, this, combined with the synergism between these compounds, may have contributed to better antiproliferative activity compared to γ-CD.

Although there are advantages in using native cyclodextrins (α-, β-, and γ-CD) to carry hydrophobic substances and form binary systems, they could have limited application. For example, water solubility, especially for β-CD with limited solubility (~18.4 mg/mL), is limited by the relative binding strength in the crystalline state [45], resulting in precipitates that may cause nephrotoxicity [16]. In addition, low complexation efficiency and the limited applicability of drug doses below 200 mg require larger amounts of CDs to solubilize the host molecules, increasing the likelihood of toxicity [46].

Therefore, the development of ternary complexes is commonly used to overcome these limitations. This multi-component system, or ternary inclusion complex, is built by merging fractions of appropriate excipients, such as hydrophilic polymers, organic acids, amino acids, and hydroxylated organic amines, into a binary complex, improving the complexation and solubilization potential of CDs, thereby optimizing cost, toxicity, and final formulation volume [47,48].

Patent No. 3 [25] presented promising quaternary formulations with propolis as active ingredient and β-CD for pharmaceutical, dermocosmetic, and dietary supplement purposes. The inventors developed four formulations, called ternary and quaternary systems, varying different solubilizing agents (natural and derived amino acids) and their respective proportions for the purpose of quantifying soluble propolis in the different systems (Table 2).

The second formulation (quaternary composition) exhibited a higher solubilization rate, between 30 and 40%, compared to Formulation 4 (tertiary composition); that is, adding a second substance to aid solubilization (ammonium glycyrrhizate) is promising for solubilizing insoluble or poorly soluble compounds.

DiCapua et al. [49] performed the coprecipitation of propolis by supercritical assisted atomization (SAA) using two carriers, HP-β-CD and polyvinylpyrrolidone (PVP), aiming to protect the bioactivity of propolis from oxidation and improve its bioavailability. The results confirmed the SAA process effectiveness. Thus, amorphous spherical particles were obtained. These SAA particles showed a polyphenol loading efficiency of up to 100% for HP-β-CD coprecipitates and up to 96% for PVP coprecipitates, with DPPH radical inhibition up to (17.2 ± 2.8) g/mL and (17.3 ± 1.0) g/mL, respectively.

Furthermore, a ternary system composed of green propolis, β-CD, and chitosan was reported for particle optimization and integration into pectin-based 3D-printable biodegradable inks. The 3D-printable inks formed transparent films after drying and exhibited rapid degradation upon contact with aqueous media. Studies of in vitro wound healing showed that incorporation of the complex into the films further improved wound healing and adhesives antimicrobial activity [50]. Thus, ternary and/or quaternary systems with CD have been obtained to improve the viability and effectiveness of the complexation and shielding process of the phenolic compounds presented in ethanolic propolis extract.

### 4.2. Propolis-in-Cyclodextrin-in-Liposomes

In Patent No. 4 [26], the innovators patented the manufacturing process of a stable system formed by a colloidal dispersion of propolis with controlled release for incorporation in cosmetic and pharmaceutical preparations and their therapeutic applications, such as their use as antimicrobial, immunostimulant, and antioxidant agents. The inventors used propolis dispersed in the solvent mixture 1,3-propanediol/water in different proportions, together with HP-β-CD or β-CD (6.45%), with stirring at 3000 rpm and a temperature of 35 °C. Then, a liposomal suspension was added in the proportion of 1.5% (*w*/*w*). The liposomal suspension was formed by different proportions and compositions of lipids such as phosphatidylcholine, phosphatidylethanolamine, lysophosphatidylcholine, phosphatidylinositol, phosphatidic acid, and cholesterol. The formulations exhibited high levels of gallic acid (>1600 mg/L) and encapsulation efficiency (>95%) and increased ATP compared to NHDF control (>100%), with PDI (<0.5) and the average particle size between 100 and 350 nm.

CDs and liposomes have the ability to create inclusion complexes with lipid-soluble drugs, encapsulated in the liposome aqueous core [51,52,53]. The liposome-loaded drug–CD complex was first developed by McCormack and Gregoriadis in 1994 [54]. This drug–CD–liposome combination represents an interesting carrier as it takes advantage of both CD and liposome carriers and aims to surmount the limitations of both systems [55]. Low concentrations of CD incorporated into the aqueous core of liposomes preserve membrane integrity without affecting liposome properties [56,57]. In addition, CD has the potential to be a cryoprotectant for liposomal membranes and also for disaccharides [58,59].

There are no studies in the scientific literature on propolis–CD–liposome. However, this system delivers other plant secondary metabolites such as curcumin [60], eucalyptol [51], α-pinene [61], limonene [53], and essential oil [62].

### 4.3. Emulsions Stabilized by Cyclodextrins

Patent No. 5 [27] dealt with water-soluble and alcohol-free propolis nanopowder development for the evaluation of antidiabetic activity. The method for preparing the nanopowder was based on first obtaining a propolis emulsion containing varying quantities of lysine (surfactant), with a ratio of 1:1 to 1:2 of concentrated propolis emulsion, sufficient for the ideal dispersion state. The prepared water-soluble propolis emulsion was pulverized using a wet grinder and then subjected to particle size analysis. Dispersed particles of 20.3 nm were identified. Then, 1 g of water-soluble propolis emulsion was mixed with β-CD (1.5 to 2 g). Afterward, the water-soluble propolis emulsion mixed with CD was frozen, dried, and pulverized. The “transmission electron microscopy” (TEM) analysis results, together with particle size analysis, revealed a particle size of 200 to 300 nm. Regarding the antidiabetic effect, the inventors noticed that the blood glucose levels of the group of animals (rats) administered with alcohol-free water-soluble propolis nanopowder decreased significantly each hour in comparison to the blood glucose levels of the group administered with propolis.

A recent line of research in the food and pharmaceutical industries is the search for identifying the capacity of substances with quality to form and stabilize emulsions for oral use [63]. Hence, selection or functional emulsions (Pickering) refer to emulsions stabilized by solid particles [64]. This phenomenon was discovered by Ramsden in 1903 and first described in 1907 [65]. Pickering emulsions, stabilized by solid particles at the oil/water interface, have garnered attention because of their enduring stability, additional functionality, and greater safety [66]. Among these particles, CDs showed appropriate size, chemical structure, human digestibility, and environmental sustainability, making them excellent stabilizers [64]. They aggregate and form crystalline nanoparticles at the oil/water interface by self-assembly, which are adsorbed on the surface of the oil droplets to maintain the long-term stability of the emulsion during the emulsification process [67,68]. This has raised great interest among researchers in recent years [69,70,71,72].

In this scenario, Fadiloglu et al. [73] studied the preservative efficacy of propolis–β-CD emulsion coated with whey protein isolate (WPI) on sea bass (*Dicentrarchus labrax* L., Moronidae) fillets stored at 4 °C. The WPI-βCD-0.2%–propolis coating yielded the best results, which extended the shelf life of sea bass fillets by 8 days, not jeopardizing their organoleptic properties.

### 4.4. Cyclodextrin-Assisted Extraction and Encapsulation

Extraction is a crucial step in the utilization of propolis bioactive compounds. Selecting the most adequate extraction method is crucial for success in obtaining high-quality and economically viable propolis products, since the extraction of propolis is a complex process because its chemical composition can vary significantly [74]. This means that an effective extraction method for one type of propolis could be inadequate for another [75]. However, all types of propolis have low solubility in water and are more easily solubilized in organic solvents, since resins are predominantly nonpolar, regardless of their chemical composition [76].

Different procedures have been cited in the literature for extracting the bioactive components of propolis, for example, maceration [77,78,79], Soxhlet extraction [80,81], ultrasonic extraction [82,83], microwave extraction [84,85], and supercritical fluid extraction [14,86,87,88]. It is worth noting that, among these, maceration is the most traditional and common method.

But due to its resinous characteristics, extraction usually has a low yield. To enhance its extraction efficiency, immersion in organic solvents for a longer period of time or at higher temperatures is used. Additionally, other techniques may require specialized equipment, but these variations in methods may not meet the requirements of large-scale production, increasing extraction costs.

For instance, in a comparative study evaluating the extraction yields of propolis using traditional and modern techniques, maceration for 72 h yielded 55–58%, while ultrasonic-assisted extraction (UAE) for 30 min yielded 41–53%. In contrast, microwave-assisted extraction (MAE) performed in only two 10 s intervals resulted in a significantly higher yield of 73–75% [89]. These results demonstrate the potential of innovative techniques to increase extraction efficiency and reduce processing time and energy consumption.

The conventional method of extracting propolis involves the use of alcohol, water, and glycerin [75]. However, alcoholic extracts in particular are poorly soluble in water, and even if they are temporarily soluble, their absorption rate is low, with a high probability of being deposited on the lining of the digestive tract. In addition, ethanol-based propolis extracts have disadvantages such as residual taste and odor and are unsuitable for use in areas such as pediatrics, ophthalmology, and otolaryngology, as well as in patients with alcohol intolerance [90].

For this reason, developing an alternative method to produce highly concentrated propolis extracts by an ethanol-free production process would expand the use of these extracts in several new areas.

Patent No. 6 [28] was aimed at extracting propolis using a new method where the propolis components form inclusion complexes during extraction. Through this approach, propolis was extracted at room temperature, without using ultrasound or microwaves, by adding HP-β-CD to the extraction solvent system (water and glycerin).

The inventors found an increase in extraction efficiency owing to the specific inclusion of propolis bioactive compounds in the HP-β-CD cavity, which increased their solubility as assessed by the measurement of antioxidant capacity parameters, including 2,2-diphenyl-l-picrylhydrazyl (DPPH) radical quenching, total phenolic content using the Folin–Ciocalteu method, and total flavonoid content, which reached values of >3.5 ± 0.5 (Trolox equivalent), >880 ± 20 (mg gallic acid), and >0.28 ± 0.02 (mM quercetin), respectively. Therefore, the advantages and innovative elements of the method were its simplicity and high efficiency, and that it did not require energy consumption, it did not depend on pH, and its high content of triols and the antimicrobial capacity of propolis made it a self-sustaining diol.

Among other emerging techniques, vacuum resistive heating extraction (VRHE) also presents enhanced extraction efficiency. Based on ohmic heating under vacuum conditions, VRHE yielded 45.72 mg GAE/g of total phenolic content in liquid propolis extract, compared to only 24.21 mg GAE/g obtained through conventional maceration (50 °C for 24 h) [91]. Furthermore, the total flavonoid content extracted using VRHE was approximately five times higher than that obtained via maceration. Despite its significant performance, this method requires heat and specialized equipment. In contrast, the CD-assisted extraction described in Patent No. 6 achieved high efficiency under milder, energy-free conditions, and with added benefits of improved solubility and stability through inclusion complex formation, underscoring its superiority in terms of simplicity, sustainability, and potential scalability.

Therefore, low-cost and efficient green methods for propolis extraction are urgently needed. According to Fernandes et al. [92], green chemistry aims to achieve higher extraction yields with reduced energy consumption, shorter processing times, and lower costs. In this context, CD-assisted extraction represents a promising approach, as it has less environmental impact compared to conventional methods that rely on organic solvents such as ethanol. This contributes significantly to the sustainability of propolis processing and aligns with the principles of green chemistry. In addition to these advantages, cyclodextrin-assisted extraction is considered an emerging green technology with great potential. CDs can act as extraction enhancers by forming inclusion complexes with natural compounds, thereby improving their extraction from plant matrices [93].

Moreover, CD encapsulation is recognized for increasing both the solubility and stability of bioactive molecules [94]. Recently, CD has been successfully used to extract bioactive compounds, like the selective extraction of glabridin from crude extracts of *Glycyrrhiza glabra* L. (Fabaceae) by SB-β-CD [95], where the yield and purity of glabridin reached, respectively, 97% and 95%. Zhang et al. [96] found that β-CD solutions resulted in higher extraction yields of petunidin-3-O-(trans-p-coumaroyl)-rutinoside-5-O-glucoside and total anthocyanins from *Lycium ruthenicum* Murr. (Solanaceae) fruits than pure water and aqueous solutions of HP-β-CD, ethanol, and methanol. It is conceivable that CD-assisted extraction ended up being a sustainable and highly effective method.

Regarding the liquid extract drying, i.e., obtaining powdered propolis extract, Silva et al. [97] introduced the propolis extract directly into a spray dryer to obtain powder. However, the resulting dried products showed limited stability owing to the absence of a protective coating process, which is key for preserving the active compounds during drying and storage.

In this context, Patent No. 7 [29] refers to a production process of water-soluble propolis powder. To obtain this product, propolis was first dissolved in hot ethyl alcohol (10–20%) and then filtered (called the first extract). After cooling the first extract, pH was adjusted to 7–10 with an alkaline solution (second extract), and α-CD and β-CD were added in the ratio 1:1:0.5 to 2 of α-CD, respectively (third extract). Finally, for the extract drying stage, starch was mixed with the third extract, and then it was lyophilized to form the powdered propolis (fourth extract).

The inventors obtained the final product with a particle size of 100 to 500 µm and a zeta potential of −50 mV or less. These results showed excellent dispersion stability and increased propolis solubility in cold water. The starch was important in the freeze-drying process to avoid the agglomeration of the propolis powder and to soften the final soluble product’s taste.

Therefore, the encapsulation method with different coating materials is rarely studied in the process of developing alcohol-free, polyphenol-rich propolis extract powder to increase its use and mask its astringency and bitterness [10,98]. The encapsulation process consists of storing an active ingredient or a substance mix, which may comprise small particles, liquid, or gaseous, in a coating or shell that provides protection for subsequent release [99,100].

Thus, various processing technologies are used in the encapsulation of bioactive compounds, with most of these techniques involving drying processes, like using a fluidized bed, spraying, supercritical fluid, and freezing [98]. Also, high-molecular-weight coating agents have been used, including carbohydrates, proteins, gums, etc. [10,40].

Regarding the encapsulation of propolis bioactive compounds, several studies have been carried out considering different high-molecular-weight materials and the physicochemical characteristics, phytochemical profile, and biological properties of the formed systems [100,101,102,103,104,105,106,107,108].

Patent No. 8 [30] refers to a process for the preparation of a skin repairing and functional emulsion containing nanopropolis extract (nanometric powder extract of propolis). The preparation process consisted of first performing the ethanolic extraction of propolis with ultrasound and then mixing with a 5% (m/m) aqueous solution of β-CD using a high-pressure homogenizer, followed by lyophilization to obtain solid particles of nanopropolis extract. The nanopropolis was then mixed with the basic components that constituted the functional repair emulsion.

The solid particles of the nanopropolis extract had a particle size between 220.1 nm and 231.4 nm, and the antioxidant activity based on the superoxide anion elimination in 180 s was increased from 90.4% to 96.5% compared to the commercially available extract. The final product (functional repair emulsion) did not show phase separation and crystallization at any temperature tested (50 °C, 25 °C, and −5 °C for 2 months), proving to be a stable emulsion.

Compared to the conventionally developed propolis, the nanopropolis extract obtained using the method described in Patent No. 7, owing to its nanometric size and the formation of an inclusion complex with β-CD during the extraction process, may show an improvement in solubility and permeability in the skin, which could intensify its biological effects, such as accelerating the regeneration of body tissues. The ultrasound-assisted biphasic extraction ensures the preservation of propolis bioactive compounds, while the combination with β-CD protects these compounds during the subsequent process stages, such as lyophilization.

Therefore, encapsulation methods using different materials and approaches have proven to be effective in improving the stability of propolis bioactive compounds, thus broadening its applicability in the food and pharmaceutical industries. Selecting the most appropriate encapsulation method and materials depends essentially on the final product’s desired properties, as well as the processing, transport, and storage conditions [109]. In addition, this approach can increase the commercial perception of propolis, which can improve the financial status of beekeepers and promote beekeeping, particularly in developing countries.

## 5. Conclusions

After this search, it was possible to perceive a comprehensive overview of the current innovations related to propolis and CD. The robust analysis enables an understanding of these technologies in the Latin American and global scenario. A low flow of patent applications and active and/or granted patents related to propolis and cyclodextrin was found. However, notable innovations were identified in the therapeutic use of propolis paired with CD in diabetes management, treatment of skin and gastrointestinal cancers, and therapeutic applications such as its use as an antimicrobial, immunostimulant, and antioxidant.

Using inclusion complexes containing propolis combined with excipients and other types of systems, such as liposomal and functional emulsions, confirms the efficacy of these associations in several pharmaceutical applications. Also, the use of cyclodextrin in the process of extracting propolis without organic solvents and obtaining powder extracts even at the nanometric scale emphasizes the application of these components and processes in the context of innovation, including the field of nanotechnology.

Thus, the literature on the complexation of propolis with CDs confirms the results of patents and shows that this strategy can significantly improve the solubility and the biological/pharmacological efficacy of propolis. This opens doors to new possibilities for improvement and obtaining new opotherapeutic products.

Nevertheless, important knowledge gaps remain. Future studies should explore the development of new cyclodextrin derivatives tailored to interact more specifically with propolis components, aiming to optimize inclusion efficiency, stability, and targeted delivery. Additionally, there is a need for the design of combined drug formulations incorporating propolis–CD complexes for synergistic effects. Investigations into in vivo pharmacokinetics, biodistribution, and toxicity are also essential to validate the clinical potential of these complexes. Advancing in these directions may enable the translation of current innovations into effective and safe pharmaceutical products.

## Figures and Tables

**Table 1:** Summary of patents found in the review.

	Application Number (Reference)	Country	Year	International Patent Classification System	Applicants	Type of Cyclodextrin	Indications or Applications
1	US 10420804 B2 [23]	US	2017–2019	A61K35/00, A61K47/00, A61P1/00, A61P35/00	MANUKA HEALTH NEW ZEALAND LTD	γ-CD	Treatment or prevention of gastrointestinal cancer
2	EP3169321 [24]	European Patent Office (EPO)	2015–2017	A61K31/216, A61K31/353, A61K9/00, A61K31/724, A61K31/192, A61K35/644	MANUKA HEALTH NEW ZEALAND LTD	α-CD β-CD γ-CD	Treatment or prevention of skin cancer
3	WO 2004/050063 A1 [25]	WIPO	2004–2007	A61K8/44, A61K8/98, A61K8/63, A61K8/73, A61K9/00, A61K14/09	ACTIMEX S.R.L	β-CD	Propolis delivered in a hydrophilic carrier
4	EP3380081 [26]	EPO	2015–2018	A61K9/127, A61K8/04, A61Q19/00	APIVITA S.A	HPβ-CD β-CD	Antioxidant, antiangiogenic, photoprotective, antimicrobial, and immunostimulant
5	KR1020100078349 [27]	South Korea	2010–2012	A61K 35/644, A61K47/40, A23L1/706, A23P1/02	GABO FARMS CO., LTD.	β-CD	Production of alcohol-free water-soluble nanopowder, and production thereof
6	GR 1007520 B [28]	Greece	2010–2012	A61K8/988, A23L21/20, A23L33/10, A61K8/738,	APIVITA KALLYNTIKA DIAITITIKA FARMAKA ANONYMI EMPORIKI KAI VIOTECHNIKI ETAIREIA	HPβ-CD	Extraction and formation of inclusion complexes of propolis active components
7	KR1020210104944 [29]	Republic of Korea	2020–2021	A61K 35/644, A23L 33/10, A23P 30/10, A61K 47/69, A61K 11/08, A61K 8/73	BNCARE AGRICULTURE CO., LTD.	α-CD β-CD	Method for manufacturing water-soluble propolis
8	CN 110664725 A [30]	China	2019–2021	A61K8/98, A61K8/73, A61K8/9789, A61K8/9794	BEIJING ZHONGMI TECH DEVELOPMENT CO LTD	β-CD	Emulsion containing nano-propolis extract

**Table 2:** Solubility study adapted from Corvi Mora et al. (2002) [25].

Formulation	Solubility
Propolis	465 μg/mL
1-Quaternary composition: propolis, ammonium glycyrrhizate, β-CD, and L-glycine (1:1:7.5:0.5 *w*/*w*)	1.695 μg/mL
2-Quaternary composition: propolis, ammonium glycyrrhizate, β-CD, and glutamic acid (1:1:7.5:0.5 *w*/*w*)	2.430 μg/mL
3-Tertiary composition: propolis, β-CD, and L-glycine (1:7.5:0.5 *w*/*w*)	1.584 μg/mL
4-Tertiary composition: propolis, β-CD, and glutamic acid (1:7.5:0.5 *w*/*w*)	1.999 μg/mL
